# Recent progress in chemoradiotherapy for oesophageal squamous cell carcinoma

**DOI:** 10.1093/jjco/hyae005

**Published:** 2024-02-10

**Authors:** Kotoe Oshima, Takahiro Tsushima, Yoshinori Ito, Ken Kato

**Affiliations:** Division of Gastrointestinal Oncology, Shizuoka Cancer Center, 1007 Shimonagakubo, Nagaizumi-cho, Sunto-gun, Shizuoka, 411-8777, Japan; Division of Gastrointestinal Oncology, Shizuoka Cancer Center, 1007 Shimonagakubo, Nagaizumi-cho, Sunto-gun, Shizuoka, 411-8777, Japan; Department of Radiation Oncology, Showa University School of Medicine, Tokyo, Japan; Department of Head and Neck, Esophageal Medical Oncology, National Cancer Center Hospital, Tokyo, Japan

**Keywords:** chemoradiotherapy, oesophageal squamous cell carcinoma, immunotherapy, clinical trials

## Abstract

Oesophageal squamous cell carcinoma is a common malignancy worldwide. Definitive chemoradiotherapy is the standard treatment for patients with resectable stage oesophageal squamous cell carcinoma who cannot undergo surgery, as well as those with locally advanced unresectable oesophageal squamous cell carcinoma. However, it has several disadvantages such as poor survival, radiation-related toxicities and severe and lethal complications related to salvage treatment for residual or recurrent disease. Numerous clinical trials on chemoradiotherapy have been conducted to confirm the optimal combination of irradiation and chemotherapy. For advanced disease, multimodal treatment strategies including salvage surgery are essential. Palliative chemoradiotherapy is also crucial for dysphagia in locally advanced oesophageal squamous cell carcinoma with or without metastatic lesions. Recently, the synergistic mechanism of radiotherapy combined with immunotherapy has been reported. Early phase clinical trials suggest that a combination of immunotherapy and chemoradiotherapy can improve clinical outcomes with manageable side effects, but further investigations are needed. Here, we reviewed the existing clinical data and current development of chemoradiotherapy combined with immunotherapy in patients with oesophageal squamous cell carcinoma.

## Teaser

Chemoradiotherapy is one of the key treatment modalities for patients with oesophageal cancer. We reviewed existing clinical data and current development of chemoradiotherapy for oesophageal squamous cell carcinoma.

## Introduction

Oesophageal cancer is the 8th most common cancer and 6th leading cause of cancer death worldwide (1). Oesophageal cancer is histologically classified into two major subtypes; squamous cell carcinoma and adenocarcinoma. Oesophageal squamous cell carcinoma (SCC) is the major histological type, accounting for 85% of all oesophageal cancer worldwide. Although oesophageal adenocarcinoma (AC) accounts for only 14% of oesophageal cancer, it is predominant in the United States and other Western countries (1,2). There are etiological, biological and clinical differences between oesophageal SCC and AC, therefore different therapeutic approaches are required (3). A previous report showed that oesophageal SCC was associated with poorer survival compared with oesophageal AC (4).

Oesophageal SCC is managed with multimodal treatments based on the disease state, such as endoscopic resection (ER), surgical resection, systemic chemotherapy and chemoradiotherapy (CRT). In particular CRT plays an important role in patients with oesophageal SCC not only for definitive treatment, but also for palliative care. The Radiation Therapy Oncology Group (RTOG) trial 85-01 demonstrated that CRT was superior to radiotherapy (RT) alone for T1-3NanyM0 oesophageal cancer (5), and thus CRT has become the standard of care. CRT has the advantages of being less invasive than surgical resection and enabling organ preservation, but it also has drawbacks such as late toxicities and severe complications after salvage treatment for residual or recurrent disease (6–8). Moreover, the survival of patients with resectable oesophageal SCC treated with definitive CRT remains poor, with 3-year survival rate of 44.7–63.8% (9). In response, various clinical trials have been conducted to improve the safety and efficacy of CRT for oesophageal SCC. Herein, we summarize the development and recent findings of CRT, as well as discuss future its perspectives including immunotherapy amongst patients with ESCC. In this review, TNM stage is described according to the Japanese Classification of Oesophageal Cancer 12th edition (10) unless otherwise specified.

## Definitive chemoradiotherapy

CRT is recognized as the standard treatment for patients with resectable oesophageal SCC who refuse surgical resection or who are poor surgical candidates (Table 1).

**Table 1:** Clinical trials investigating chemoradiotherapy for stage I/II/III/IVA (T1-3NanyM0-1a) oesophageal cancer

Clinical trial	Stage Histology	Regimen	Radiation dose (Gy)	CRR (%)	OS (%)	Ref.
JCOG9708	Stage I SCC	CRT with 5FU 700 mg/m^2^ (day 1–4, 29–32); and cisplatin 70 mg/m^2^ (day 1, 29)	60	87.5	4-year 80.5	11
JCOG0502	Stage I SCC	CRT arm: with 5FU 700 mg/m^2^ (day 1–4, 29–32); and cisplatin 70 mg/m^2^ (day 1, 29)	60	87.3	5-year 85.5	12
JCOG0508	Stage I SCC After ER	Without residual disease Prophylactic CRT arm: with 5FU 700 mg/m^2^ (day 1–4, 29–32); and cisplatin 70 mg/m^2^ (day 1, 29)	41.4	–	3-year 90.7	18
JCOG9906	Stage II/III SCC	CRT with 5FU 400 mg/m^2^ (day 1–5, 8–12, 36–40, 43–47); and cisplatin 40 mg/m^2^ (day 1, 8, 36, 43)	60	62.2	3-year 44.7	19
RTOG95-04	Stage II/III SCC, AC	Standard irradiation arm/higher irradiation arm; in both arms, with 5FU 1000 mg/m^2^ (day 1–4, 29–32); cisplatin 75 mg/m^2^ (day 1, 29)	50.4/64.8	NR/NR	2-year 40 2-year 31	22
JCOG0909	Stage II/III SCC	CRT with 5FU 1000 mg/m^2^ (day 1–4, 29–32); and cisplatin 75 mg/m^2^ (day 1, 29)	50.4	58.5	3-year 74.2	23
ARTDECO	Stage I-IVA SCC, AC	Standard irradiation arm/higher irradiation arm: in both arms, with paclitaxel 50 mg/m^2^ and carboplatin AUC 2 (day 1, 8, 15, 22, 29, 36)	50.4/61.6	NR	3-year 42/39	24
CONCORDE/ PRODIGE-26	Stage I-III SCC, AC	Standard irradiation arm/higher irradiation arm: in both arms, with oxaliplatin 85 mg/m^2^, leucovorin 200 mg/m^2^ and bolus 5FU 400 mg/m^2^ (day 1, 15, 29); infusional 5FU 1600 mg/m^2^ (day 1–3, 15–17, 29–31)	50/66	NR	NR (median, 25.2/23.5 months)	25
NCT01937208	Stage II-IVA SCC	Standard irradiation arm/higher irradiation arm; in both arms, with docetaxel 25 mg/m^2^ and cisplatin 25 mg/m^2^ (day 1, 8, 15, 22, 29)	50/60	26/25	3-year 52.7/53.1	26
NCT02850991	Stage II-IVB	Standard irradiation arm/higher irradiation arm: in both arms, with paclitaxel 50 mg/m^2^ and carboplatin AUC 2 (day 1, 8, 15, 22, 29, 36)	50.4/59.4	NR	3-year 26.0/28.1	27
PRODIGE5/ ACCORD17	Stage I-IVA SCC, AC	5FU + cisplatin arm: 5FU 1000 mg/m^2^ (day 1–4, 29–32); and cisplatin 75 mg/m^2^ (day 1, 29) FOLFOX arm: in both arms, with oxaliplatin 85 mg/m^2^, leucovorin 200 mg/m^2^ and bolus 5FU 400 mg/m^2^ (day 1, 15, 29); infusional 5FU 1600 mg/m^2^ (day 1–3, 15–17, 29–31)	50 50	43 44	3-year 26.9 3-year 19.9	29
JCOG0604	Stage II/III SCC	S-1 60 mg/m^2^ (day1–14, 29–42) and cisplatin 75 mg/m^2^ (day 1, 29)	50.4	59.5	3-year 61.9	35

Abbreviations: CRR, complete response rate; OS, overall response; Ref, reference; SCC, squamous cell carcinoma; AC, adenocarcinoma; CRT, chemoradiotherapy; NR, not reported; ER, endoscopic resection.

### Stage I (T1N0M0)

In the Japan Clinical Oncology Group (JCOG) 9708, a phase II trial, definitive CRT consisting of cisplatin and 5-fluorouracil (CF) with 60 Gy of RT demonstrated a complete response (CR) rate of 87.5% (63/72 patients) and a 4-year survival rate of 80.5% with mild toxicity (11). Based on these results, the JCOG0502 trial was conducted to demonstrate non-inferiority of CRT (60 Gy RT plus CF) to surgery for stage I disease. A total of 368 patients (surgery group: *n* = 209; CRT group: *n* = 159) were enrolled in the nonrandomized part of the trial (12). The 5-year survival rates were 86.5% and 85.5% in the surgery and CRT groups, respectively (adjusted hazard ratio [HR] 1.05; 95% confidence interval [CI] 0.67–1.64). CRT was non-inferior to surgery in terms of overall survival (OS), the primary endpoint, in clinical T1bN0M0 oesophageal SCC. In contrast, the 5-year progression free survival (PFS) rate was lower in the CRT group versus the surgery group (71.6% vs. 81.7%: adjusted HR 1.48; 95% CI 1.01–2.16). Although the CR rate in the CRT group was 87.3% (138/158 patients), regional recurrence was more frequently observed in the CRT group (33/48 patients, 68.8%) than in the surgery group (17/32 patients, 53.1%). This regional recurrence in CRT group could be attributed to the radiation field in this trial targeting only the primary lesion without elective nodal irradiation (ENI). Furthermore, 23% of the CRT group, underwent surgery or ER as salvage treatment for residual or recurrent disease. Salvage surgery after CRT has been associated with increased treatment-related morbidity and mortality (7,8). A randomized trial (JCOG1904) is ongoing to verify the superiority of modified CRT (50.4 Gy) with ENI, and it is expecting a reduction of locoregional recurrence versus conventional CRT (60 Gy) without ENI for the clinical T1bN0M0 patients (13).

Although surgery or definitive CRT is recommended for patients with clinically stage I oesophageal SCC, patients with T1a disease have the potential to be cured with minimally invasive treatment, such as ER alone (14). It is often difficult to clearly differentiate between clinical T1a and T1b. ER allows for the evaluation of histopathological depth of tumour invasion and lymphovascular invasion, which are useful to predict the risk of local recurrence and lymph node metastasis (15–17). A prospective trial (JCOG0508) showed the effectiveness of diagnostic ER for clinical T1b (submucosal 1–2) followed by selective CRT based on histologic evaluation (18). This provides a new non-invasive treatment strategy.

### Stage I/II/III/IVA (T1-3NanyM0-1a)

Twenty years ago in Japan, definitive CRT with wide-field, 60 Gy radiation therapy was the standard treatment for patients with resectable advanced oesophageal SCC who refused surgery, based on the JCOG9906 trial (19). The regimen in this trial were considered tolerable, but 5.3% of treatment-related deaths due to late toxicities were reported. Another retrospective study also showed that eight out of 78 patients (10.2%) without cancer recurrence died of cardiopulmonary toxicity after CRT (6). These late toxicities might be caused by the high irradiation dose and the extended field of radiation. In contrast, 14 out of 26 patients with residual or locally recurrent tumour received salvage treatment (19). There was no operative mortality or hospital death in this trial, but irradiation doses >55 Gy were associated with increased postoperative mortality and morbidity (20).

In Western countries, standard radiation dose for definitive CRT is 50.4 Gy (21), because a higher irradiation dose (64.8 Gy) did not improve survival and local control to the standard dose (50.4 Gy) in INT 0123 (RTOG 94-05) (22). The JCOG0909 trial was conducted to confirm the efficacy of CRT modifications including ENI and salvage treatment, aiming to reduce CRT-related toxicities and facilitating salvage treatment for improved survival (23). CF therapy plus concurrent RT of 50.4 Gy was delivered. Good responders received 1–2 additional cycles of chemotherapy. For residual or recurrent disease, salvage ER or surgery was performed immediately after CRT or additional chemotherapy based on specific criteria. CR was achieved in 55 patients (59%), whilst salvage ER and surgery were performed in five (5%) and 25 (27%) patients, respectively. Grade 3–4 early operative complications were observed in five patients (20%), with one (4.0%) treatment-related death. Grade 3 late toxicities were observed in 9 patients (9.6%) but there were no grade 4 or 5 late toxicities. The 3-year OS was 74.2%, confirming the study hypothesis. The modification in both radiation dose and radiation fields in CRT, as well as the optimal timing of salvage treatment after failure of CRT, might have contributed to the prolonged survival and fewer treatment-related toxicities.

The development of radiation techniques, including intensity modulated radiation therapy (IMRT), 3-dimensional conformal RT (3D-CRT) and volumetric modulated arc therapy has improved sparing of surrounding tissues, which may permit a higher local radiation dose without increasing toxicity. Several randomized clinical trials on irradiation dose escalation or radiation field using modern radiation techniques have been conducted to improve locoregional control. The ARTDECO study compared high-dose (61.6 Gy) versus standard-dose (50.4 Gy) CRT for medically inoperable and/or unresectable oesophageal cancer including both squamous cell carcinoma and adenocarcinoma. There was no significant improvement in PFS across different histologic types (24). The CONCORDE (PRODIGE-26) study was another randomized study in which standard-dose (50 Gy) was compared with an escalated dose (66 Gy) CRT. ENI was delivered in both groups. No benefit in locoregional PFS with escalated dose was shown (25). Moreover, Chinese randomized trials comparing high-dose versus standard-dose IMRT also failed to show improvement of local/regional failure-free survival and overall survival with dose-escalated CRT (59.4–60 Gy) compared with standard-dose CRT (50–50.4 Gy) for locally advanced oesphageal SCC (26,27).

Having an optimal radiation field is also critical for improving efficacy and reducing toxicities. One meta-analysis demonstrated that involved-field irradiation (IFI) showed a significant improvement in OS at 5 years and a significant decrease in grade ≥3 acute oesophagitis versus ENI for patients with oesophageal cancer who underwent definitive radiotherapy and CRT (28). When comparing the types of radiotherapy across subgroups, IFI had a significant advantage in 5-year OS versus ENI in the IMRT subgroup, whereas no differences were observed in the 3D IMRT–mixed (both 3D-CRT and IMRT) subgroup. In terms of toxicity, the IMRT subgroup had no difference in the incidence of grade ≥3 acute oesophagitis between ENI and IFI. Further investigations of efficacy and safety during long-term follow-up are required for each radiotherapy technique.

Combination chemotherapy with CF is a widely accepted standard regimen for CRT, but it requires prolonged intravenous hydration, continuous 5FU infusion and good renal function. Other combinations with concurrent irradiation have been studied, such as the FOLFOX (5FU plus leucovorin and oxaliplatin) regimen, which was evaluated in the PRODIGE5/ACCORD17 trial that included 85% patients with stage I-IVA eesophageal SCC (29). Although CRT with FOLFOX did not increase PFS compared with CRT with CF, FOLFOX is recognized as an alternative to CF in combination with RT due to its convenience and less renal toxicity. A combination of paclitaxel (PTX) and carboplatin (CBDCA) is the standard regimen for neoadjuvant CRT in resectable oesophageal cancer (30), and this has also been used as definitive treatment (31). Although there is no large phase III study that directly compares this regimen with CF, conflicting results on efficacy have been reported in retrospective and observational studies (31–34). Meanwhile, the combination of S-1 and cisplatin with CRT was evaluated in JCOG0604, a phase I/II trial. The replacement of 5FU to S-1 did not show improved efficacy for stage II/III oesophageal SCC (35).

### Stage IVA (T4NanyM0-1a)

CRT is the only treatment with a potentially curative intent for patients with unresectable locally advanced oesphageal SCC without distant metastasis (i.e. clinical T4 and/or unresectable regional lymph node metastasis), with a dose of 60 Gy is as the standard regimen according to the JCOG0303 trial (36). In the JCOG0303 trial, low-dose CRT did not have improved efficacy and toxicity profiles over standard-dose CRT. Nevertheless, the prognosis in patients with unresectable locally advanced oesophageal SCC remains poor (median survival time: 13.1 months, 3-year OS: 25.9% in the standard-dose CRT group).

A phase I/II study of induction chemotherapy (ICT) followed by CRT showed promising results. Amongst patients with unresectable locally advanced oesophageal SCC who received definitive CRT after ICT consisting of docetaxel and CF (DCF), the CR rate was 39.4%, PFS was 12.2 months and OS was 26.0 months (37). Nevertheless, there are some critical issues related to CRT in patients with unresectable locally advanced oesophgeal SCC. First, fistula formation was observed in 23% (32/140 patients) during or after treatment in the standard-dose CRT group in the JCOG0303 trial (36). Fistula formation is associated with massive bleeding and pneumonia; it can be the primary cause of treatment-related death. ICT may reduce the incidence of fistula formation. Second, oesophageal stenosis due to CRT might cause a decrease in quality of life even amongst patients who achieved CR after CRT. Previous report showed that palliative intervention for dysphagia was more frequently required in patients who treated with CRT alone compared with CRT followed by surgery for locally advanced thoracic oesophageal cancer (38). Third, salvage oesophagectomy after CRT has been associated with high rates of treatment-related morbidity and mortality, even for resectable oesophageal SCC, although surgery was associated with better survival and local control (7,39). It remains unclear whether surgery benefits the survival of patients with residual lesions after definitive CRT for initially unresectable locally advanced disease, but surgery is a common clinical practice for residual lesions in Japan. The COSMOS trial investigated the safety and efficacy of chemoselection with ICT with DCF and subsequent conversion surgery for initially unresectable locally advanced oesophageal SCC (40). Conversion surgery was performed in 41.7% (20/48 patients), and R0 resection was achieved in 39.6% (19/48 patients). The estimated 1-year OS was 67.9% with no serious postoperative complications. The JCOG1510 trial is ongoing to confirm the superiority of induction DCF plus conversion surgery or definitive CRT versus definitive CRT without ICT for OS in patients with locally advanced, initially unresectable oesophageal SCC (41).

## Preoperative (neoadjuvant) chemoradiotherapy

The European Society for Medical Oncology clinical practice guidelines state that preoperative CRT, not preoperative chemotherapy, consisting of weekly CBDCA and PTX with 41.4 Gy RT is recommended as the standard of care for locally advanced resectable oesophageal SCC based on the results of the Chemoradiotherapy for Esophageal Cancer Followed by Surgery Study (CROSS) (21,30). In Japan, preoperative chemotherapy with CF had been the standard treatment for patients with resectable oesophageal SCC based on the results of the JCOG9907 (42). Evidence from Western countries cannot be applied to the Asian population due to differences in histology, operative indication, and the procedure of oesophagectomy. The JCOG1109 trial was conducted to investigate the superiority of CRT using CF (CF-RT), as well as of DCF, over chemotherapy alone with CF as preoperative therapy for locally advanced, resectable oesophageal SCC (43). The CF-RT group, compared with the CF group, had higher rates of R0 resection and pathological CR (98.9% vs. 90.3%, 36.7% vs. 2.2%, respectively), but PFS and OS were similar (median 7.0 vs. 5.6 years: HR 0.84; 95%CI 0.63–1.12, *P* = 0.12). In the CF-RT group, however, distant recurrence and non-cancerous death were more common, and RT and CRT were less frequently used during subsequent therapy after recurrence. These differences between the CF-RT and CF groups may affect the survival outcome. Similarly, previous studies showed increasing postoperative mortality and no significant benefit in overall survival despite better tumour response with neoadjuvant CRT (44,45). In contrast, DCF showed superiority over CF in terms of PFS (median not reached vs. 5.6 years: HR 0.67; 95%CI 0.51–0.88, *P* = 0.004) and OS (median not reached vs. 5.6 years: HR 0.68; 95%CI 0.50–0.92, *P* = 0.006). Thus, DCF represents a new standard preoperative treatment for locally advanced oesophageal SCC in Japan (46).

Recently, the SANO-trial reported that neoadjuvant CRT followed by active surveillance showed non-inferior OS at 2 years versus neoadjuvant CRT followed by surgery. At least 35% of patients undergoing active surveillance were spared from non-beneficial oesophagectomy (47). Active surveillance of oesophageal cancer after response to neoadjuvant CRT may be a new treatment strategy for organ preservation in patients with resectable oesophageal cancer. The CROC trial proposed another strategy for organ preserving. In the trial, DCF was administered for patients with resectable advanced oesophageal SCC as ICT. Then, definitive CRT was delivered for patients with remarkable response, whereas the others underwent surgery. Amongst patients who underwent ICT followed by CRT, 89.8% (44/49) achieved CR, and 1-year organ preservation survival in the whole population (*n* = 90) was 56.8% (48). Although preoperative DCF chemotherapy followed by surgery is now the standard treatment for locally advanced, resectable oesophageal SCC in Japan (46), the CROC trial suggested that CRT for good responders after ICT can preserve the oesophagus.

## Palliative chemoradiotherapy

### Stage IVB (TanyNanyM1)

Most patients with oesophageal massive tumours suffer from dysphagia, which causes nutritional compromise and decreases of quality of life (49). Although palliative chemotherapy is the recommended standard treatment for incurable patients (50), it is difficult to palliate dysphagia unless the tumour shrinks. Several treatment modalities are available for the palliation of dysphagia, such as external-beam RT, CRT, intraluminal brachytherapy, endoluminal stent placement and bypass surgery (51,52). Previous reports showed that palliative RT was associated with an equivalent relief of moderate to severe dysphagia over time and a decreased risk of adverse effects such as fistula, perforation and haemorrhage compared with stent placement (53). TROG 03.01, a phase III randomized trial, demonstrated that palliative CRT, versus RT alone, showed a modest, but non-significant, increase in dysphagia relief rate (45% vs. 35%), dysphagia-PFS (median: 4.1 vs. 3.4 months) and OS (median: 6.9 vs. 6.7 months) versus RT alone. Grade 3–4 acute toxicity was significantly more frequent in the CRT group than the RT group (36% vs. 16%) (54). In a retrospective study of patients with clinical stage IVA/B oesophageal SCC, palliative CRT using CF with an RT of 40 Gy in 20 fractions was associated with a significantly improved dysphagia score versus palliative RT alone of 30 Gy in 10 fractions (51). The most appropriate treatment for malignant dysphagia remains unclear, but palliative CRT might be a crucial modality for the palliation of dysphagia.

## Chemoradiotherapy with immune checkpoint inhibitors

There has been significant progress in the development of immune checkpoint inhibitors (ICIs) that promote the anti-tumour activity of T cells by inhibiting immune checkpoints such as programmed death 1 (PD-1), programmed death ligand 1 (PD-L1) and cytotoxic T lymphocyte associated antigen 4 (CTLA-4). Immunotherapy, specifically ICI monotherapy, dual combination of ICIs and ICIs plus cytotoxic agents, showed clinical benefit for metastatic or recurrent oesophageal SCC patients (55). This promoted the development of combination treatments of ICIs and RT or CRT. RT reportedly has synergistic effects with immunotherapy (56,57). Irradiation enhances local anti-tumour immune responses by increasing tumour antigen release, promoting immune cell recruitment and modulating the expression of immune checkpoint molecules. Moreover, RT is expected to induce an immune-mediated abscopal effect (57). Therefore, the combination of CRT plus immunotherapy is considered as an attractive strategy.

In a phase II trial (NCT02844075), patients with resectable oesophageal SCC received pembrolizumab (anti-PD-1) combined with neoadjuvant CRT for 5 weeks followed by surgery. Postoperatively, patients were treated with pembrolizumab for 2 years. Oesophagectomy was performed in 26 out of 28 patients. There were two morbidities due to acute lung injury after surgery. The pathologic CR in the primary tumour was 46.1%, with 6- and 12-month OS rates of 89.3% and 82.1%, respectively (58). The addition of pembrolizumab to neoadjuvant CRT in oesophageal SCC was suggested to be efficacious.

Numerous clinical trials are being conducted to investigate the efficacy and safety of immunotherapy combined with definitive or neoadjuvant CRT for oesopahgeal SCC patients (Table 2), and these are expected to have better clinical outcomes (59–71). Nevertheless, it is still necessary to monitor and manage immune-related adverse events because unexpected side effects or additive toxicities could potentially occur (57).

**Table 2:** Clinical trials investigating the combination of chemoradiotherapy and immunotherapy in oesophageal cancer

Clinical trial	Phase	Histology	No. of Pts	Regimen	CRR (%)	OS	Ref.
Neoadjuvant therapy							
NCT02844075	II	SCC	28	CRT + pembrolizumab → postoperative pembrolizumab	pCR 46.1	1-year, 82.1%	58
NCT03792347 (PALACE-1)	IB	SCC	20	CRT + pembrolizumab	pCR 55.6	NR	59
NCT02998268	II	AC	40	Chemotherapy with or without pembrolizumab → CRT + pembrolizumab	NR (major pathological response, 50.0)	NR	60
NCT03044613	II	AC SCC	16	Cohort A; induction nivolumab → CRT + nivolumab	37.5	NR	61
NCT03490292	I/II	AC SCC	22	CRT + avelumab	pCR 26	NR	62
NCT04437212	II	SCC	20	CRT + toripalimab → postoperative toripalimab	pCR 54	NR	63
NCT04973306 (iCROSS)	II/III	SCC	176	CRT + tisrelizumab vs. CRT	NR	NR	–
NCT05357846	III	SCC	422	CRT + sintilimab vs. CRT	NR	NR	–
Definitive therapy							
NCT03222440	Ib	SCC	20	CRT + camelizumab (max 32 week)	cCR 11	Median, 16.7 months	64
NCT03377400	II	SCC	40	CRT + durvalumab + tremelimumab → durvalumab (max 2 year)	NR	2-year, 75%	65
UMIN000034373 (TENERGY)	II	SCC	50	CRT + atezolizumab (max 1 year)	cCR 42.1%	Median, 31.0 months; 1 year, 65.8%	66
NCT04005170	II	SCC	42	CRT + toripalimab (max 1 year)	cCR 62	Median, not reached; 1-year, 78.4%	67
NCT03437200 (CRUCIAL)	II	SCC AC	130	CRT + nivolumab or nivolumab + ipilimumab (max 1 year)	NR	NR	–
NCT04210115 (KEYNOTE-975)	III	SCC AC	600	CRT + pembrolizumab (max 1 year) vs. CRT + placebo	NR	NR	68
NCT03957590 (RATIONALE311)	III	SCC	366	CRT + tislelizumab (max 2 year) vs. CRT + placebo	NR	NR	69
NCT04550260 (KUNLUN)	III	SCC	600	CRT + durvalumab (max 2 year) vs. CRT + placebo	NR	NR	70
NCT04543617 (SKYSCPAPER-07)	III	SCC	750	CRT → Tiragolumab + Atezolizumab (max 17 cycles) vs. Atezolizumab + placebo (max 17 cycles) vs. double placebos	NR	NR	71

Abbreviations: CCR, complete response rate; OS, overall survival; Ref, reference; SCC, squamous cell carcinoma; AC, adenocarcinoma; CRT, chemoradiotherapy; pCR, pathological complete response; cCR, clinical complete response; NR, not reported.

## Conclusion and future direction

Definitive CRT is clinically indicated for patients with resectable oesophageal SCC who do not consent to surgery or are poor surgical candidates, as well as for patients with locally advanced unresectable oesopahgeal SCC. Several clinical trials have explored various treatment strategies with CRT modification, aiming to further improve survival and tolerability, as well as reduce toxicity. Currently, clinical trials testing ICIs containing CRT are underway for resectable and unresectable locally advanced oesophageal SCC. ICIs are expected to enter clinical practice of definitive CRT as in metastatic and recurrent oesophageal SCC. Further investigations, including biomarker analysis, which can predict treatment response and resistance are needed to optimize the treatment strategy for oesophageal SCC.

## Funding

None to be declared.

## Conflict of interest statement

Ken Kato has received research funds from Ono Pharmaceuticals, Bristol Myers Squibb, MSD, Merck Biopharma, Taiho Pharmaceutical, Bayer, AstraZeneca, Janssen and Oncolys Biopharma; and honoraria from Ono Pharmaceuticals, Bristol Myers Squibb, MSD and Taiho Pharmaceutical.
